# Gut microbiota associations with chronic kidney disease: insights into nutritional and inflammatory parameters

**DOI:** 10.3389/fmicb.2024.1298432

**Published:** 2024-05-21

**Authors:** Vladimir Lazarevic, Daniel Teta, Menno Pruijm, Catherine Stoermann, Nicola Marangon, Julie Mareschal, Raquel Solano, Arlene Wurzner-Ghajarzadeh, Nadia Gaïa, Patrice D. Cani, Oğuzhan S. Dizdar, François R. Herrmann, Jacques Schrenzel, Laurence Genton

**Affiliations:** ^1^Genomic Research Laboratory, Geneva University Hospitals and University of Geneva, Geneva, Switzerland; ^2^Nephrology, Hospital of Sion, Sion, Switzerland; ^3^Nephrology, University Hospital of Lausanne and University of Lausanne, Lausanne, Switzerland; ^4^Nephrology, Geneva University Hospitals and University of Geneva, Geneva, Switzerland; ^5^Department of Nephrology, Geneva University Hospitals and Clinique of Champel, Geneva, Switzerland; ^6^Clinical Nutrition, Geneva University Hospitals and University of Geneva, Geneva, Switzerland; ^7^Metabolism and Nutrition Research Group, Louvain Drug Research Institute, UCLouvain, Université catholique de Louvain, Brussels, Belgium; ^8^WELBIO-Walloon Excellence in Life Sciences and Biotechnology, WELBIO Department, WEL Research Institute, Wavre, Belgium; ^9^Department of Internal Medicine and Clinical Nutrition Unit, Kayseri City Training and Research Hospital, University of Health Sciences, Kayseri, Türkiye; ^10^Rehabilitation and Geriatrics, Geneva University Hospitals and University of Geneva, Geneva, Switzerland; ^11^Infectious Diseases, Geneva University Hospitals and University of Geneva, Geneva, Switzerland

**Keywords:** appetite, gut permeability, handgrip strength, hormones, lean body mass, lipopolysaccharide, Metataxonomics 16S, nutritional state

## Abstract

**Introduction:**

The gut barrier, comprising gut microbiota, plays a pivotal role in chronic kidney disease (CKD) progression and nutritional status. This study aimed to explore gut barrier alterations in hemodialyzed (HD) patients, non-HD (NHD) CKD patients, and healthy volunteers.

**Methods:**

Our cross-sectional study enrolled 22 HD patients, 11 NHD patients, and 11 healthy volunteers. We evaluated fecal microbiota composition (assessed via bacterial 16S rRNA gene sequencing), fecal IgA levels, surrogate markers of gut permeability, serum cytokines, appetite mediators, nutritional status, physical activity, and quality of life.

**Results:**

HD patients exhibited significant alterations in fecal microbiota composition compared to healthy volunteers, with observed shifts in taxa known to be associated with dietary patterns or producing metabolites acting on human host. In comparison to healthy volunteers, individuals with HD patients exhibited elevated levels of inflammatory markers (CRP, IL-6 and TNF-α), glucagon-like peptide-2, and potential anorexigenic markers (including leptin and peptide YY). NHD patients had increased levels of CRP and peptide YY. Overall fecal microbiota composition was associated with height, soft lean mass, resting energy expenditure, handgrip strength, bone mineral content and plasma albumin and TNF-α.

**Discussion:**

Compared to healthy volunteers, HD patients have an altered fecal microbiota composition, a higher systemic inflammation, and a modification in plasma levels of appetite mediators. While some differences align with previous findings, heterogeneity exists likely due to various factors including lifestyle and comorbidities. Despite limitations such as sample size, our study underscores the multifaceted interplay between gut microbiota, physiological markers, and kidney function, warranting further investigation in larger cohorts.

## Introduction

Chronic kidney disease (CKD) has a worldwide prevalence of 10 to 16% (Kazancioğlu, 2013). End-stage renal disease, which refers to an estimated glomerular filtration <15 mL/min/1.73m^2^ (Vassalotti et al., 2016), is generally treated with dialysis. Over 2.5 million of patients receive renal replacement therapy and this number is expected to double within 10 years. A low glomerular filtration rate is related to complications such as protein-energy wasting (PEW), decreased physical function, cardio-vascular diseases, pulmonary edema, anemia, bone disease, and neuropathy (National Kidney Foundation, 2000). As PEW is highly prevalent and increases the risk of hospitalization and mortality (Huang et al., 2010; Mak et al., 2011), the National Kidney Foundation recommends routine assessment of the nutritional state in patients with end-stage kidney disease through measurements of body weight and composition, laboratory parameters, and nutritional intakes (National Kidney Foundation, 2019).

The gut barrier could be involved in the pathogenesis of CKD and its complications. It is secured by the gut epithelium and its tight junctions, the mucus, the gut-associated lymphoid tissue, and the microbiota (Magnotti and Deitch, 2005). Especially the gut microbiota encounters an increased interest in the scientific community. It can synthesize and/or metabolize uremic toxins (Popkov et al., 2022; Krukowski et al., 2023) which are substances that accumulate in the blood in case of CKD and have been related to cardio-vascular diseases and CKD progression (Vanholder et al., 2018). Thus, the gut microbiota composition and function may influence the outcome of CKD patients and be a therapeutic target (Sumida et al., 2021). Several studies suggest alterations of gut microbiota in CKD. In rats, uremia leads to gut dysbiosis, with a decreased abundance of Lactobacillaceae and Prevotellaceae (Vaziri et al., 2013a), and a disruption of intestinal tight junctions (Vaziri et al., 2013b). Cross-sectional human studies, summarized in a recent review, found alterations of the fecal microbiota composition and its metabolites in patients with advanced CKD, whether hemodialyzed (HD) or not, as compared to healthy persons (Chung et al., 2019). Most of these studies did not evaluate other parameters of the gut barrier and did not differentiate the impact of hemodialysis *per se* from uremia, although it may also affect the gut microbiota (Luo et al., 2021). If hemodialysis is associated with additional alterations in gut microbiota, the treatment of CKD progression and complications by gut microbiota modulation may be different in HD than in non-dialyzed patients with CKD (termed NHD) patients.

The first aim of the study was to compare the gut barrier and microbiota of HD patients, NHD, and healthy volunteers. We hypothesized that HD patients have an altered composition of fecal microbiota, an increased gut permeability and systemic inflammation, and an imbalance of plasma appetite mediators in favor of anorexigenic mediators, compared to the other groups. The second aim was to evaluate the associations between fecal microbiota and clinical and biological parameters, including nutritional parameters.

## Methods

This cross-sectional analysis took place in the University Hospitals of Geneva and Lausanne, the Clinic of Champel Geneva, and the Hospital of Sion between August 1st 2016 and August 31st 2019. The protocol was accepted by the local Ethical Committees, registered under clinicaltrials.gov (NCT 02962089), and all participants signed an informed consent.

### Study population

This post-hoc analysis encompasses three groups of subjects, i.e., 22 HD patients, 11 NHD patients and 11 healthy volunteers.

The inclusion criteria for the HD patients were maintenance HD ≥3 months and absence of systemic antibiotics for an acute infection in the previous month. This group consisted of 11 patients with features of PEW at screening (plasma albumin <38 g/L, or body weight loss >5% of dry body weight over the last 3 months; daily dietary intakes <30 kcal/kg and < 1 g protein/kg as calculated by 24 h dietary recall), recruited for an interventional nutritional study (Genton et al., 2021), and 11 patients without these features. Initially, the four groups (HD patients with PEW, without PEW, NHD patients and healthy volunteers) were matched for age (± 5 years) and sex. Features of PEW were assessed at screening. However, there were no significant differences in albumin and dietary intakes at the time of measurement between groups (Friedman ANOVA: albumin, Friedman test = 10.761, *p* = 0.376; dietary intakes, Friedman test = 7.500, *p* = 0.6775). Thus, we decided to pool the data of HP patients in a single group, which led to the loss of pairing. The NHD patients included non-dialyzed patients with CKD stage 4 or 5 with daily dietary intakes >30 kcal/kg as calculated by 24 h dietary recall. The healthy volunteers were included based on the following criteria: body mass index <30 kg/m^2^, absence of chronic disease potentially leading to wasting such as chronic infections, cancer, rheumatoid arthritis, congestive cardiomyopathy, end stage renal disease, chronic obstructive disease, cystic fibrosis, Crohn’s disease, or alcoholic liver disease.

The exclusion criteria were the same for all participants: cognitive impairment, life expectancy <1 year, enteral or parenteral nutrition, inadequate dialysis defined by sKt/V < 1.2 (if applicable), decreased plasma albumin levels related to liver failure or exudative enteropathy, nutritional supplements containing fibers since <1 month, drugs influencing body composition since <1 month (systemic corticosteroids, insulin, testosterone, post-menopausal hormone therapy, injectable contraceptives), known endocrinological disorder leading to hypo- or hypermetabolism untreated or treated since <1 month, pregnancy and breast-feeding.

### Measurements

We assessed clinical and routine biological parameters in the fasted state, as well as nutritional state, systemic inflammatory status, serum levels of appetite mediators, surrogate markers of gut permeability and fecal microbiota. The parameters of the HD patients with PEW were measured up to 6 weeks after screening, to allow them enough reflection time for their participation in the longitudinal study and for the organization of their baseline tests. The fecal microbiota composition and the biological parameters related to the study were determined in the University Hospitals of Geneva at the end of the study, while the routine biological parameters were performed in the local laboratories.

### Clinical parameters

The weight and height were measured without shoes and in light clothes, immediately after dialysis for the HD participants, to calculate body mass index. Soft lean mass, bone mineral content, and fat mass were assessed by dual-energy x-ray absorptiometry (Hologic Discovery A^®^, Hologic, Waltham, MA, United States). In HD patients, this measurement was performed within 90 min of the end of the hemodialysis.

Energy and protein intakes of two weekdays and one day of the weekend were assessed through a 3-day food diary filled in by the patient and reviewed with a dietician. Resting energy expenditure was assessed after an overnight fast by indirect calorimetry (Quark RMR^®^, Cosmed, Pavone, Italy). Appetite was evaluated by a visual analog scale of 100 mm, ranging from 0 (no appetite) to 100 mm (excellent appetite) (Suneja et al., 2011).

Handgrip strength was measured with a hydraulic hand dynamometer (Baseline^®^ 12–0240, White Plains, New York, USA), thrice with each hand. For analysis, we used the maximum value obtained from both hands (Leong et al., 2016).

Physical activity was assessed with a pedometry device worn at the waist for 7 consecutive days (Yamax Digiwalker SW-200^®^, London, United Kingdom) (Crouter et al., 2003; Schneider et al., 2003). For analysis, we considered the mean number of steps per day.

Quality of life was assessed with the RAND 36-item short form health survey (RAND Health Care, 2023). It evaluates eight health domains, each scoring from 0 (very unfavorable) to 100% (very favorable) (RAND Health Care, 2023): physical functioning, limitations due to physical health, limitations due to emotional health, energy/fatigue, emotional well-being, social functioning, pain, and general health.

### Biological parameters

All participants underwent measurements of venous hemoglobin, prealbumin, urea, creatinine, bicarbonate, albumin, pre-albumin, and C-reactive protein (pre-dialysis if applicable). In the setting of this research, we additionally assessed the serum levels of cytokines, hormones involved in appetite regulation, and surrogate markers of gut permeability as glucagon-like peptide-2 (GLP-2) and lipopolysaccharides (LPS) (Szeto et al., 2018; Genton et al., 2021). ELISA allowed the determination of serum levels of interleukin (IL)-6, IL-10, tumor necrosis factor (TNF)-α, leptin, total ghrelin, total glucagon-like peptide-1, peptide YY (U-Plex metabolic group assays; MSD, Rockville, MD, USA), cholecystokinin (Antibodies-online, Aachen, Germany), GLP-2 and neuropeptide Y (Merck, Darmstadt, Germany) and active grehlin (Gentaur, Kampenhout, Belgium). Serum LPS levels were assessed by competitive inhibition enzyme immunoassay (Genton et al., 2021). Fecal IgA levels were measured by ELISA according to the instruction of the manufacturer (IBL International, Hamburg, Germany).

### Fecal microbiota composition

Stool collection: The patients collected a nut-sized sample of their feces into Feces Tube (Sarstedt, Nürmbrecht, Germany), stored them in their fridge at 2–8°C, and transported them to the laboratory within 24 h. Stool samples were then aliquoted for fecal IgA measurements into 2 mL safe-lock Eppendorf tubes. The remaining material in the Feces Tube was used for DNA extraction. All tubes were kept frozen at −80°C. Processing of the samples occurred at the end of the study.

DNA extraction: DNA was extracted from 125 to 230 mg stool samples using ZymoBIOMICS DNA Miniprep Kit (Zymo Research, Irvine, CA, United States). Purified DNA was quantified using the Qubit dsDNA BR Assay Kit (Thermo Fisher Scientific, Carlsbad, CA, United States) and stored at −20°C. Three negative extraction controls (NECs) were performed previously (Genton et al., 2021) by extracting DNA using the same extraction procedure but omitting the addition of stools.

Amplicon sequencing: The V3–4 region of the bacterial 16S rRNA genes (positions 341–805 in *Escherichia coli* 16S rRNA gene) was amplified using 3 ng of extracted DNA in a 25 μL volume of ZymoTaq PreMix (Zymo Research) containing each of 0.4 μM forward primer 341F 5’-CCTACGGGNGGCWGCAG-3′ and reverse primer 805R 5’-GACTACHVGGGTATCTAAKCC-3′. The PCRs were carried out with an initial denaturation at 95°C for 10 min, 29 cycles at 95°C for 30 s, 51°C for 30 s and 72°C for 60 s, followed by a final extension at 72°C for 7 min. For each sample, duplicate PCRs were combined and run on a 2100 Bioanalyzer (Agilent Technologies, Santa Clara, CA, United States) for quality analysis and quantification. The amplicon barcoding/purification, library construction, 2 × 300 Illumina (San Diego, CA, USA) MiSeq sequencing, and library demultiplexing were performed as described previously (Lazarevic et al., 2016).

Sequence analysis: Paired reads were joined using PEAR v0.9.11 (-m 470 -n 390 -v 10 -p 0.0001 -u 0) (Zhang et al., 2014). Merged sequence reads were clustered into zero-radius operational taxonomic units (zOTUs) using UNOISE3 (Edgar, 2016) from USEARCH v10.0.240 package (Edgar, 2010). The zOTUs were defined using 181 samples and 3 negative controls collected during the interventional study (108 of which were analyzed in a previous contribution (Genton et al., 2021)). From zOTUs with >90% identity to reference EzBioCloud 16S database (Yoon et al., 2017) sequences (as of 19 August 2019) as revealed by USEARCH (-id 0.90 -query_cov 0.99), we removed those with <10 reads in the whole dataset (*n* = 784), as well as those represented with >100x higher average relative abundance in NECs than in samples (n = 12). Remaining zOTUs (*n* = 2,201) were classified using EzBioCloud 16S database via MOTHUR (Schloss et al., 2009) (command classify.seq with the options method = wang and cutoff = 80). Sequencing data were submitted to European Nucleotide Archive (ENA; study number: PRJEB49241). Sequencing data from the 9 of 11 here analyzed HD patients with PEW have been previously submitted [PRJEB43505 (Genton et al., 2021)]. For the 44 samples analyzed, a total of 3,543,950 raw reads were obtained. After merging forward and reverse reads, zOTU clustering, removal of putative contaminant and low abundance zOTUs as well as zOTUs with no reasonable similarity to reference 16S rRNA sequences (see above), the dataset contained 3,358,342 (merged) reads with a median (per sample) of 78,015 (range 40,031–121,019).

### Statistical methods

#### Sample size

The determination of the sample size was based on the study of Vaziri et al. (2013a). They showed significant differences in fecal microbiota when comparing 12 healthy volunteers and 24 HD patients, who were not matched for gender. At the time when the study was set up, no methodology was available to calculate sample size using microbiota as primary endpoint.

#### Non-metataxonomic data

Results are shown as median (IQR) or n (frequency). Continuous parameters were compared between the HD patients, NHD patients and healthy volunteers with Kruskal-Wallis H tests. Significance was set at *p* < 0.05 and corrected for multiple comparisons by the Benjamini-Hochberg method (Benjamini and Hochberg, 1995). In case of significance, the groups were compared two by two with the Wilcoxon rank sum tests.

#### Metataxonomic data

Principal coordinates analysis (PCoA) and hierarchical clustering (group average linking method) were performed to visualize variations in bacterial communities. These analyses, relying on Bray–Curtis similarity (Bray and Curtis, 1957) matrix, were based on the square-root-transformed relative abundance, and were performed in PRIMER (PRIMER-e, Auckland, New Zealand). We used permutational multivariate analysis of variance (PERMANOVA; 9,999 permutations) (Anderson, 2001) to assess the significance of microbiota differences and the PERMDISP (9,999 permutations) test to evaluate the homogeneity of multivariate dispersion among groups (Anderson, 2006).

Ecological indices (richness and Shannon diversity index (Shannon, 1948)) were calculated from the relative abundance of zOTUs after rarefying the dataset to the same sequencing depth (40,000) using the *rrarefy* function in the vegan v2.6–2 R v4.2.0 package. Values were compared with Wilcoxon rank sum tests.

The differences between groups in the relative abundance of individual taxa was tested using MaAsLin2 (Microbiome multivariate association with linear models) (Mallick et al., 2021). We used the default linear model (LM) with LOG transformation and filtered relative abundance (minimum 0.001%) and prevalence (minimum 25%). Benjamini-Hochberg corrected *p*-values <0.05 were considered significant.

Distance-based linear model (DISTLM, PRIMER) was used to analyze the association between the bacterial community profiles and continuous non-metataxonomic variables in the whole study population. In case of significant associations, we reformatted the symbols in the PCoA plots to visualize differences in bacterial communities after dichotomizing the subjects according to the median values of the relevant variables. The involved zOTUs were identified by Spearman tests.

## Results

We included 22 white HD patients (6 female and 16 male), 11 NHD patients (3 female and 8 male) and 11 volunteers (3 female and 8 male), thus a similar proportion of males and females in each group. The etiologies of CKD in HD and in non-dialyzed patients were mostly hypertension (73 and 82%, respectively) and diabetes (50 and 27% respectively), and often multifactorial. The etiology of CKD for each patient and the drugs against hypertension and diabetes are shown for each patient in the Supplementary Tables 1, 2, respectively.

### Non-metataxonomic data

Table 1 shows the clinical characteristics of the participants. The body composition and handgrip strength by sex is shown in Supplementary Table 3. After correction for multiple comparisons, HD patients had lower appetite rating, physical activity and general health than healthy volunteers, but their clinical characteristics did not significantly differ from NHD patients. Table 2 highlights the abnormal routine blood parameters in HD and NHD patients, as expected. Compared to healthy volunteers, HD patients had higher serum levels of inflammatory makers (CRP, IL-6, TNF-α), GLP-2, and of some possible anorexigenic (leptin, peptide YY) and orexigenic mediators (Neuropeptide YY). Compared to NHD patients, HD patients demonstrated higher levels of CRP, LPS and peptide YY.

**Table 1:** Clinical characteristics of the participants [median (interquartile range)].

	HD patients	NHD patients	Healthy volunteers	H test^*^	*p* ^*^
	*n* = 22 (6 female, 16 males)	*n* = 11 (3 females, 8 males)	*n* = 11 (3 females, 8 males)		
Age (years)	63.2 (14.3)	62.6 (17.5)	58.6 (21.5)	0.022	0.989
Height (cm)	168.6 (15.0)	170.0 (9.8)	173.0 (10.2)	1.082	0.583
Body weight (kg)	83.3 (25.7)	76.2 (16.5)	78.7 (16.9)	1.986	0.370
Body mass index (kg/m^2^)	29.9 (6.8)	25.4 (7.2)	26.0 (5.3)	5.682	0.058
Soft lean mass (kg)	53.7 (18.2)	49.3 (13.0)	49.9 (13.6)	0.221	0.895
Bone mineral content (kg)	2.4 (0.7)	2.1 (0.9)	2.3 (0.6)	1.030	0.599
Fat mass (kg)	26.3 (12.4)	23.3 (6.3)	23.9 (10.9)	3.503	0.174
Lean mass index (kg/m^2^)	18.5 (3.8)	17.2 (2.0)	18.1 (2.5)	1.565	0.457
Fat mass index (kg/m^2^)	9.6 (3.6)	7.7 (4.5)	8.1 (3.0)	4.474	0.107
Energy intake (kcal/kg)	20.3 (7.6)	26.7 (10.3)	27.8 (12.8)	5.779	0.056
Protein intake (g/kg)	0.9 (0.5)	1.1 (0.2)	1.0 (0.7)	1.133	0.567
Appetite rating (mm)	7.0 (1.0) ^a^	8.0 (3.0) ^a^	8.0 (2.0)	13.291	0.002
Resting energy expenditure (kcal)	1561.5 (644.0)	1563.0 (508.0)	1763.0 (494.0)	2.861	0.239
Handgrip strength (kg)	23.5 (11.0)	34.0 (23.0)	36.0 (12.0)	7.007	0.030
Pedometry (steps/d)	3037.2 (4061.9)^a^	5604.2 (8244.4)	5936.8 (4045.3)	10.667	0.005
General health (0 to 100%)	47.5 (25.0) ^a^	40.0 (20.0) ^a^	80.0 (25.0)	17.890	<0.001

HD: Hemodialysed patients; NHD: non-hemodialyzed patients with chronic kidney failure; Body mass index = weight (kg)/height (m)^2^; Lean mass index = (soft lean mass + bone mineral content)/height (m)^2^.

* Kruskal-Wallis H tests, Chi-squared corrected for ties with 2 degrees of freedom. With the Benjamini–Hochberg method, significance was corrected to *p* < 0.026.

a Significantly different from healthy volunteers; ^b^significantly different from NHD patients (Wilcoxon rank sum tests).

**Table 2:** Biological characteristics of the participants [median (interquartile range)].

	HD patients	NHD patients	Healthy volunteers	H test^*^	*p* ^*^
	*n* = 22 (6 female, 16 males)	*n* = 11 (3 females, 8 males)	*n* = 11 (3 females, 8 males)		
*Routine parameters*					
Hemoglobin (g/L)	113.0 (14.0)^a^	120.0 (14.0)^a^	144.0 (14.0)	22.837	<0.001
Urea (mmol/L)	21.7 (7.5)^a^	24.9 (11.5)^a^	6.0 (2.3)	24.802	<0.001
Creatinin (umol/L)	632.5 (235.0)^a^^,^ ^b^	332.0 (202.0)	84.0 (29.0)	33.669	<0.001
Bicarbonate (mmol/L)	22.0 (2.8)^a^	22.8 (5.4)^a^	26.7 (4.1)	19.706	<0.001
Albumin (g/L)	41.0 (2.0)^a^^,^ ^b^	47.0 (3.0)	45.0 (5.0)	22.786	<0.001
Prealbumin (mg/L)	325.0 (63.0)	399.0 (105.0)^a^	289.0 (98.0)	8.727	0.013
C-reactive protein (g/L)	6.2 (8.50) ^a^^,^ ^b^	1.5 (2.2)	1.4 (1.0)	23.942	<0.001
*Intestinal permeability*					
Serum lipopolysaccharides (ng/mL)	64.4 (40.1)^b^	37.4 (39.5)^a^	97.0 (30.5)	18.513	<0.001
Serum glucagon-like peptide 2 (ng/mL)	10.0 (4.4)^a^	8.3 (5.0)^a^	2.8 (0.8)	24.203	<0.001
*Systemic inflammation*					
Interleukin-6 (pg/mL)	1.4 (1.2)^a^	0.7 (0.8)	0.6 (0.6)	13.436	0.001
Interleukin-10 (pg/mL)	0.1 (0.1)	0.1 (0.2)	0.1 (0.1)	0.033	0.983
Tumor necrosis factor-α (pg/mL)	3.0 (1.6)^a^	2.4 (1.0)	1.5 (1.2)	17.124	<0.001
Fecal IgA (μg/mL)	1369.0 (4363.0)	738.0 (2800.0)	1200.0 (1725.0)	0.627	0.731
*Appetite mediators*					
Total ghrelin (pg/mL)	838.2 (403.0)	737.0 (713.9)	689.4 (581.1)	2.581	0.275
Active ghrelin (fmol/mL)	9.2 (12.8)	9.9 (8.3)	9.7 (11.4)	0.090	0.956
Leptin (pg/mL)	32643.4 (65775.8)^a^	20431.9 (53039.3)	10272.3 (10923.2)	9.518	0.009
Active glucagon-like peptide 1 (pM)	0.4 (1.0)^b^	0.1 (0.2)	0.1 (0.3)	6.764	0.034
Cholecystokinin (pg/mL)	360.7 (153.6)^a^	328.4 (161.9)	407.9 (87.4)	7.910	0.019
Neuropeptide Y (pg/mL)	61.9 (57.9)^a^	57.5 (30.0)	29.5 (15.5)	11.096	0.004
Peptide YY (pg/mL)	229.6 (107.9)^a^^,^^b^	121.7 (109.9)	54.3 (35.2)	27.672	<0.001

HD: hemodialyzed patients; NHD: non-hemodialyzed patients with chronic kidney failure. All parameters were measured in the blood besides the fecal IgA concentrations.

* Kruskal–Wallis H-tests, Chi-squared corrected for ties with 2 degrees of freedom. With the Benjamini–Hochberg method, significance was corrected to *p* < 0.026.

a Significantly different from healthy volunteers.

b Significantly different from NHD patients (Wilcoxon rank sum tests).

### Metataxonomic data

**Figure 1:**
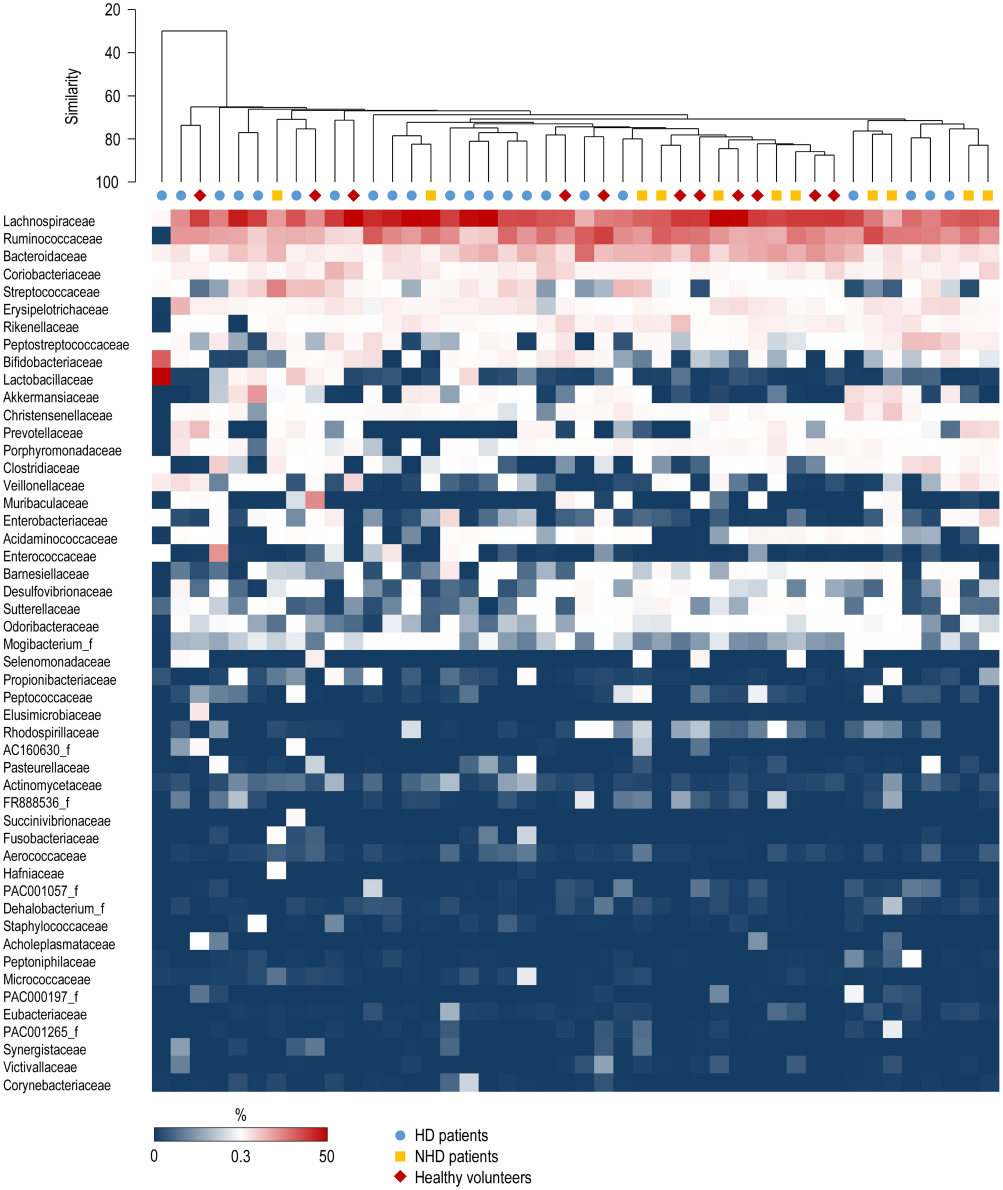
Variation in relative abundances of bacterial families among patients and control subjects. Each column corresponds to an individual patient. The top 50 families with the highest mean relative abundance are presented. The relative abundance scale is provided below the plot. The group average hierarchical clustering of samples is represented on the top. HD, hemodialyzed patients; HV, healthy volunteers; NHD, non-hemodialyzed patients with chronic kidney disease.

**Figure 2:**
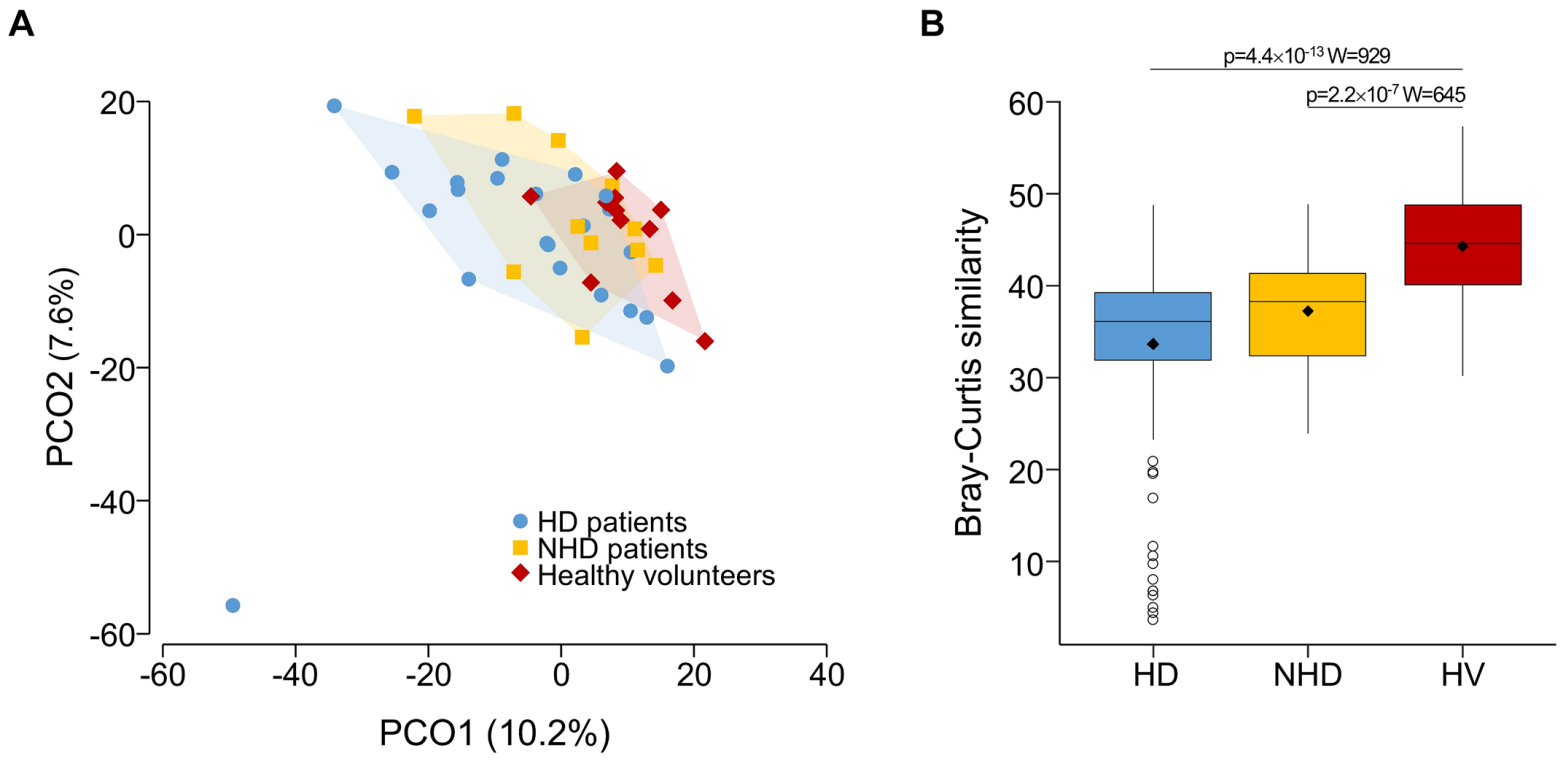
**(A)** PCoA plot of the fecal microbiota (zOTU-level) samples from hemodialysis (HD) patients, non hemodialyzed patients with chronic kidney disease (NHD) and healthy volunteers. It shows a trend of separation between the three groups. For clearer visual differentiation, the regions encompassing samples from each group (excluding one sample from the HD group) are shaded. **(B)** Bray–Curtis similarity analysis at the zOTU level depicting the similarity of individuals within each of the three studied groups. HV, healthy volunteers. Statistical analysis was performed using a Wilcoxon rank sum test.

The most abundant bacterial families constituting the gut microbiota were Lachnospiraceae, Ruminococcaceae, and Bacteroidaceae, which are typical of gut microbiota. Figure 1 provides a summary of the variation in bacterial taxonomic profiles across individuals and studied groups. The PCoA plot revealed a trend in differentiating microbiota from healthy volunteers and patients (Figure 2A). Notably, a substantial proportion of HD samples were situated outside the area formed by healthy volunteer samples, and vice versa. PERMANOVA analysis further confirmed significant dissimilarities between HD patients and healthy volunteers (*p* = 0.0038, R^2^ = 0.0506, *F* = 1.6923). The PCoA plot implies that the observed significance in the PERMANOVA test is likely attributed, at least in part, to differences in community composition, despite a distinction in multivariate dispersion between HD patients and healthy volunteers (PERMDISP *p* = 0.0037). Bray–Curtis similarity analysis underscored the highest homogeneity of the fecal microbiota in healthy volunteers and the lowest in HD patients (Figure 2B).

**Figure 3:**
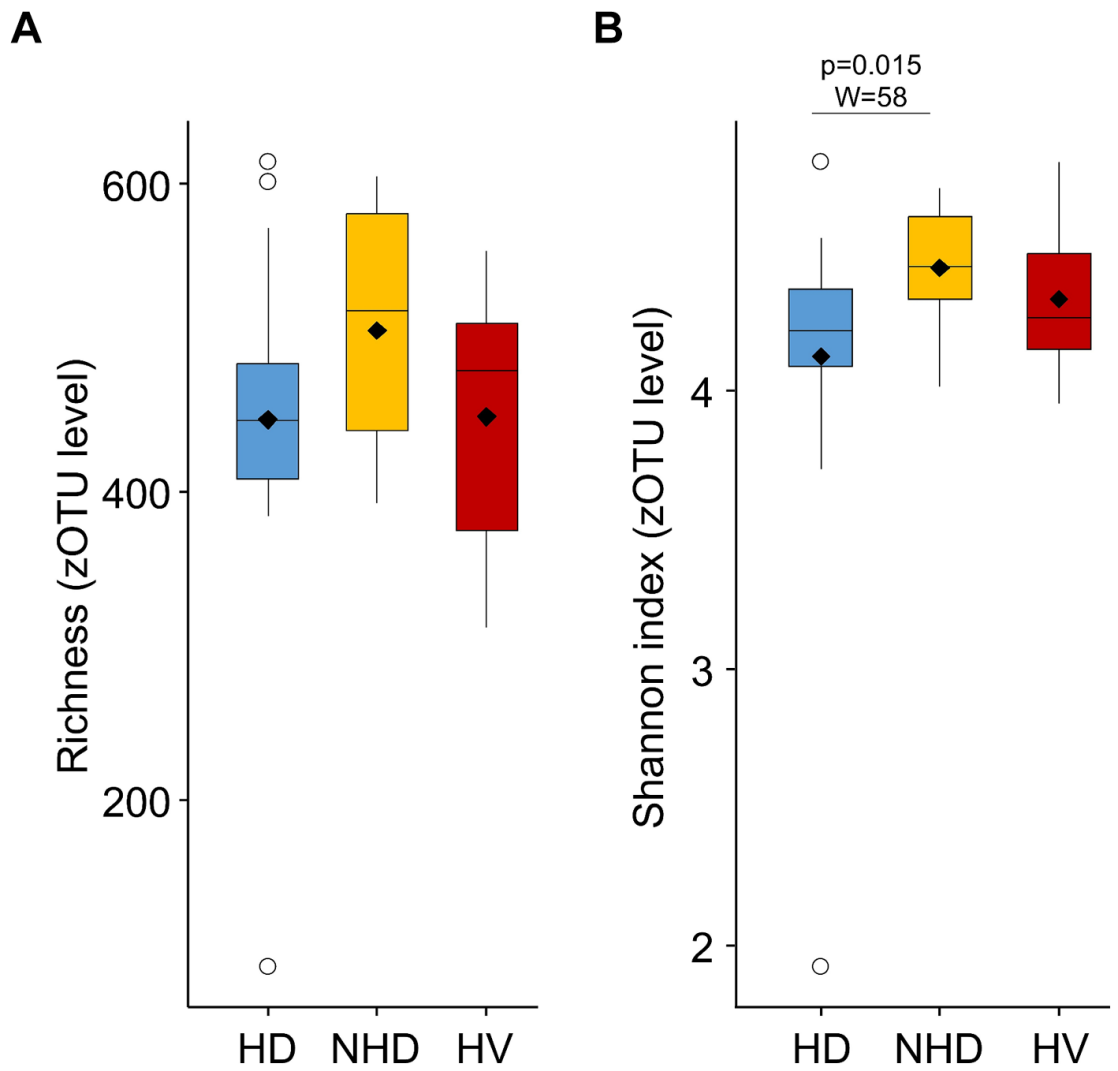
Richness **(A)** and Shannon diversity **(B)** index calculated from the relative abundance of zOTUs in hemodialysis (HD) patients, non-hemodialyzed patients with chronic kidney disease (NHD) and healthy volunteers (HV).

Visually, zOTUs richness and diversity seemed to be similar across the groups, although Shannon diversity index was significantly higher in the NHD patients compared to HD patients (Figure 3).

The MaAsLin2 analysis identified a larger number of differentially abundant zOTUs and higher-level taxa between HD and healthy volunteers in comparison to the differences between HD and NHD patients or NHD and healthy volunteers (Supplementary Table 4). Regarding short-chain fatty acid generating bacteria, we observed a decreased level of the unclassified *Oscillibacter* in NHD and HD versus heathy volunteers, and an increase in *Dialister invisus* in NHD patients as compared to healthy volunteers or HD patients. Eubacteriaceae had higher levels in both HD and NHD patients compared to healthy volunteers. *Clostridium scindens*, a bacterium involved in bile acid metabolism, exhibited higher levels in individuals with HD compared to healthy volunteers. However, after adjusting for multiple comparisons, none of the differences among groups remained statistically significant.

**Figure 4:**
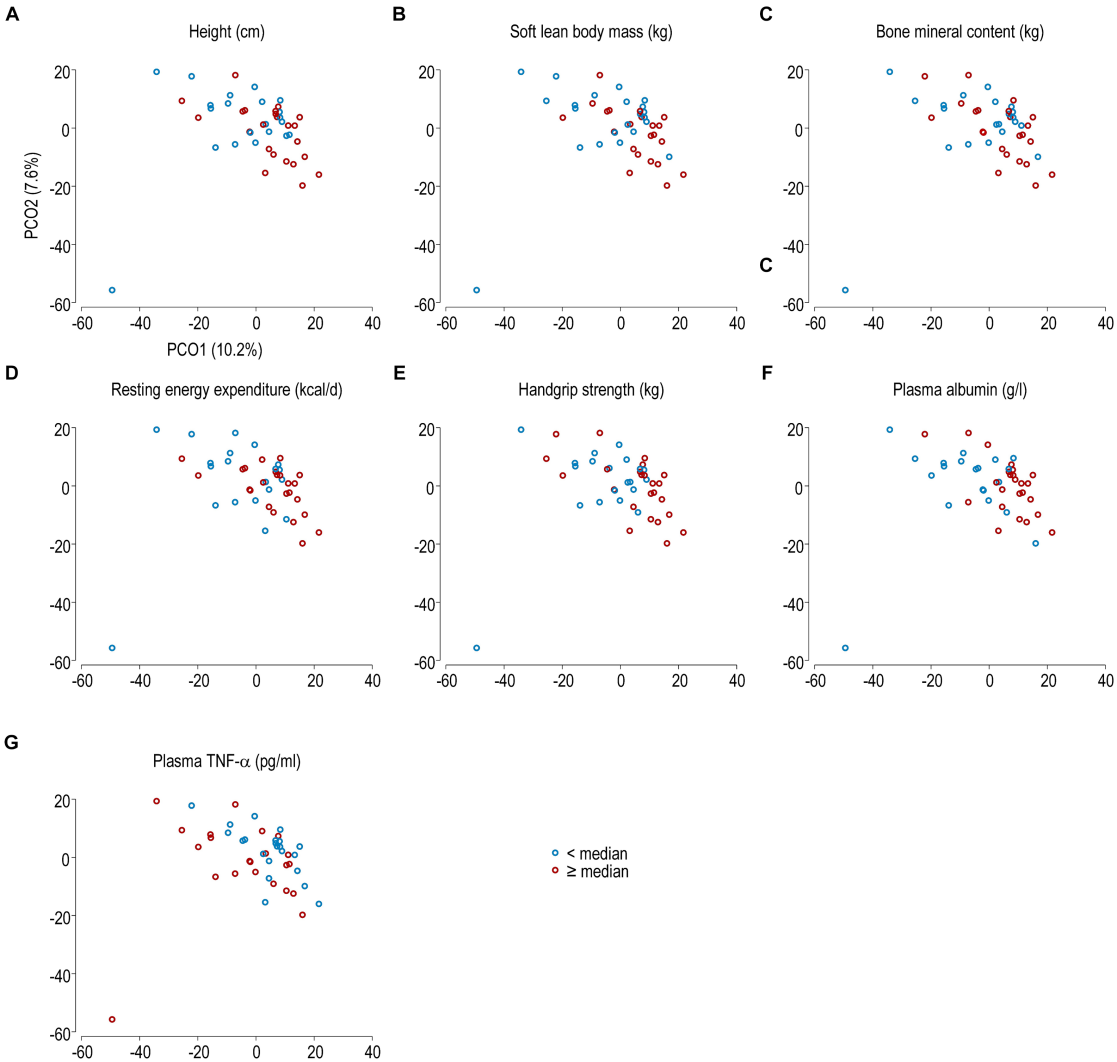
PCoA plot of the fecal microbiota samples at the zOTU level from all subjects. The subjects were categorized according to the median values of height **(A)**, soft lean body mass **(B)**, bone mineral content **(C)**, resting energy expenditure **(D)**, handgrip strength **(E)**, plasma albumin **(F)**, and plasma levels of TNF-α **(G)**. Bacterial communities of subjects with values below the median value are shown in blue, and the others in red.

The overall zOTU composition was associated with height, soft lean mass, bone mineral content, resting energy expenditure, handgrip strength, and plasma levels of albumin and TNF-α (Table 3). The median values of these parameters in the whole study population were 170.0 (162.8–175.0) cm, 51.2 (32.9–66.1) kg, 2.3 (1.9–2.5) kg, 1,618 (917–2,445) kcal/day, 30.0 (11.0–50.0) kg, 43.0 (41.0–46.0) g/L, and 2.4 (0.5–5.7) pg/mL, respectively. The zOTUs associated with these variables are shown in Supplementary Table 5. Visually, the fecal microbiota communities seemed different between individuals dichotomized according to soft lean mass, resting energy expenditure, handgrip strength, and plasma albumin (Figure 4).

**Table 3:** Associations between overall zOTU-level microbiota composition and continuous variables by DISTLM.

Variables	*p*
*Clinical characteristics*	
Height (cm)	0.0020^a^
Body weight (kg)	0.1200
Body mass index (kg/m^2^)	0.6943
Soft lean body mass (kg)	0.0006^a^
Bone mineral content (kg)	0.0046^a^
Fat mass (kg)	0.8312
Lean body mass index (kg/m^2^)	0.0572
Fat mass index (kg/m^2^)	0.4346
Energy intake (kcal/kg)	0.5390
Protein intake (g/kg)	0.4954
Appetite rating (mm)	0.1353
Resting energy expenditure (kcal/d)	0.0001^a^
Handgrip strength (kg)	0.0006^a^
Pedometry (steps/d)	0.1287
General health (0 to 100%)	0.1726
*Biological characteristics*	
Hemoglobin (g/L)	0.0150
Urea (mmol/L)	0.2579
Creatinin (umol/L)	0.0172
Bicarbonate (mmol/L)	0.1671
Albumin (g/L)	0.0052^a^
Prealbumin (mg/L)	0.6997
C-reactive protein (g/L)	0.0191
Serum lipopolysaccharides (ng/mL)	0.2754
Serum glucagon-like peptide 2 (ng/mL)	0.0259
Interleukin-6 (pg/mL)	0.0238
Interleukin-10 (pg/mL)	0.4803
Tumor necrosis factor-α (pg/mL)	0.0006^a^
Fecal IgA (μg/mL)	0.8555
Total ghrelin (pg/mL)	0.0213
Active ghrelin (fmol/mL)	0.2032
Leptin (pg/mL)	0.2856
Active glucagon-like peptide 1 (pM)	0.7765
Cholecystokinin (pg/mL)	0.2978
Neuropeptide Y (pg/mL)	0.0344
Peptide YY (pg/mL)	0.0384

a Significant after correction for multiple testing.

## Discussion

This study showed that HD patients, as compared to healthy volunteers, display alterations in overall fecal microbiota composition and specific taxa, have higher blood levels of GLP-2, of inflammatory markers, and of several possible anorexigenic mediators, and impaired appetite, physical activity and general health. NHD patients also tended to show alterations in the gut barrier, although to a smaller extent than HD patients. Overall zOTU composition was significantly associated with several nutritional and inflammatory parameters.

A few cross-sectional studies have previously compared the fecal microbiota of adult patients with end-stage renal disease and healthy volunteers. At the phylum level, several studies described a higher proportion of Proteobacteria, Actinobacteria and Firmicutes (Vaziri et al., 2013a; Chao et al., 2023), and a lower abundance of Bacteroidetes (Hu et al., 2020; Wu et al., 2023) in HD patients. Although we could not confirm these differences, we found that the overall fecal microbiota composition was significantly altered in HD patients. The differences at the genus and OTU level were heterogeneous between the published studies, likely because of confounding factors like lifestyle, genetics, co-morbidities as for instance diabetes, and treatments including hemodialysis. In our study, the protein and calorie intakes were not significantly different between groups and likely do not explain the gut microbiota differences. The HD and NHD patients were supplemented in micronutrients, advised to limit their dietary intake of sodium, and, if necessary, of other electrolytes as recommended by the National Kidney Foundation (Ikizler et al., 2020). The dietary restriction in potassium and acid through reduced intake of fruits and vegetable decreases the supply of fibers which may unfavorably affect the abundance and function of saccharolytic bacteria. Whether these dietary adaptations were indeed performed in our patients has not been determined.

Some studies report a depletion in short-chain fatty acid producing-bacteria in NHD patients vs. healthy controls (Wong et al., 2014; Jiang et al., 2016; Margiotta et al., 2020). We found a depletion in the butyrate-producer *Oscillibacter* species, known to be low also in diabetic patients (Wu et al., 2020). In contrast to other findings (Lau et al., 2021), the acetate- and propionate- producer *Dialister invisus* (Louis and Flint, 2017) was higher in our NHD patients. This anti-inflammatory bacterum has been associated with a dietary pattern rich in fruits, vegetables and fish (Shi et al., 2022). However, we did not evaluate long-term dietary patterns in our NHD patients and healthy volunteers. There is also an increasing interest in bacteria generating uremic toxins, such as p-cresyl sulfate for instance, which are associated with impaired prognosis. In our study, the abundance of Eubacteriaceae was higher in HD and NHD patients compared to the healthy controls. These bacteria of the Clostridia class, already described in patients with progressing IgA nephropathy (De Angelis et al., 2014), are known to produce p-cresyl sulfate (Gryp et al., 2017). Thus, their high levels may reflect severity of CKD. *Clostridium scindens*, the bacterial species with the ability to transform primary bile acids into secondary bile acids and convert glucocorticoids into androgens (Ridlon et al., 2013), was found to be more abundant in HD patients than in healthy controls. There is evidence of an association between the presence of this bacterium and the induction of remission in pediatric Crohn’s disease through dietary interventions (Wang et al., 2019).

Regarding gut permeability, a cross-sectional study showed that circulating LPS, a surrogate marker of gut permeability, increased with worsening of the kidney function, correlated positively with inflammation, and was highest in patients who recently started HD (McIntyre et al., 2011). In contrast, Wong et al. found that the intestinal permeability, assessed by sugar absorption tests, was not influenced by the dialysis procedure itself (Wong et al., 2019). Other studies which assessed gut permeability through different methods reported a higher gut permeability of NHD patients as compared to healthy controls (Magnusson et al., 1991; Wang et al., 2012). In our study, we found that LPS levels were not higher in HD and NHD patients, suggesting that LPS changes in these patients may not be responsive enough to assess gut permeability. The high serum levels of active GLP-2 in HD and NDH patients, a gut-trophic hormone produced by enteroendocrine intestinal L cells, was unexpected. It could represent an adaptative response to increased intestinal permeability, as high circulating levels of GLP-2 have been described in case of intestinal injury (Drucker, 2002).

Patients with CKD were reported to have higher plasma levels of CRP, IL-6 and TNF-α than controls (de Vinuesa et al., 2006; Borges et al., 2016). We could confirm the higher inflammatory markers in HD patients but not in the NHD patients. However, inflammation appears not to occur in all patients with CKD and is promoted mostly by the severity of kidney disease and the dialysis procedure (Cobo et al., 2018).

Levels of several possible anorexigenic hormones, such as leptin and peptide YY (Bossola et al., 2006), were higher in HD and NHD patients as compared to healthy volunteers, which is in line with their lower appetite. We would have also expected a lower level of Neuropeptide Y and a higher level of cholecystokinin in these patients. A recent review reports controversial results regarding plasma levels of leptin and ghrelin in patients with CKD (Wang et al., 2022). Thus, the explanation of low appetite in HD and uremia seems more complex than an imbalance of plasma appetite mediators.

Previous studies including NHD patients could not associate fecal microbiota composition with muscle mass and function (Margiotta et al., 2020, 2021). However, bacterial uremic toxins have been linked positively with inflammation (Borges et al., 2016), and negatively with body mass index and dietary intakes (Hu et al., 2022). As a novelty, we report an association of the overall microbiota composition with several nutritional parameters, as for instance soft lean mass and handgrip strength, and inflammatory parameters (C-reactive protein, IL-6, TNF-α, albumin), but these results need confirmation in larger studies.

Finally, the low physical activity and compromised health status in HD patients are not surprising as they have been described by others (Avesani et al., 2012; Zhou et al., 2018). A low handgrip strength has been linked with a low glomerular filtration rate, and a low muscle and fat mass (Zhou et al., 2018). Our HD patients tended to have a lower handgrip strength than healthy volunteers. The unexpectedly similar soft lean mass index in HD patients compared to healthy volunteers could be due to their slightly higher body mass index.

The strength of this study were the comparisons of several gut barrier components in HD, NHD patients and healthy volunteers of similar age. Our study was limited by a relatively small population but our primary endpoint was a difference in fecal microbiota composition between groups which had been demonstrated in such small populations (Vaziri et al., 2013a).

## Conclusion

Compared to healthy volunteers, HD patients have an altered composition of fecal microbiota, a higher systemic inflammation, and higher plasma levels of two anorexigenic appetite mediators, namely leptin and peptide YY. Overall bacterial composition was significantly associated with several nutritional and inflammatory parameters, but this association should be confirmed in larger studies.

## Data availability statement

The datasets presented in this study can be found in online repositories. The names of the repository/repositories and accession number(s) can be found at: https://www.ebi.ac.uk/ena, PRJEB49241; https://www.ebi.ac.uk/ena, PRJEB43505.

## Ethics statement

The studies involving humans were approved by Commission cantonale d’éthique de la recherche CCER (Geneva, Switzerland) and Commission cantonale d’éthique de la recherche sur l’être humain CER-VD (Lausanne, Switzerland). The studies were conducted in accordance with the local legislation and institutional requirements. The participants provided their written informed consent to participate in this study.

## Author contributions

VL: Conceptualization, Formal analysis, Methodology, Writing – original draft, Writing – review & editing. DT: Conceptualization, Data curation, Investigation, Methodology, Project administration, Writing – review & editing. MP: Data curation, Investigation, Methodology, Project administration, Writing – review & editing. CS: Investigation, Methodology, Writing – review & editing. NM: Investigation, Writing – review & editing. JM: Data curation, Investigation, Methodology, Project administration, Writing – review & editing. RS: Investigation, Project administration, Writing – review & editing. AW-G: Investigation, Project administration, Writing – review & editing. NG: Formal analysis, Methodology, Writing – review & editing. PC: Methodology, Writing – review & editing. OD: Writing – review & editing. FH: Conceptualization, Data curation, Formal analysis, Methodology, Writing – review & editing. JS: Formal analysis, Methodology, Project administration, Writing – review & editing. LG: Conceptualization, Data curation, Formal analysis, Funding acquisition, Investigation, Methodology, Project administration, Writing – original draft, Writing – review & editing.
