# Supplementary material for: Gut microbiota associations with chronic kidney disease: insights into nutritional and inflammatory parameters
Source: Front Microbiol. 2024 May 21;15:1298432. doi: 10.3389/fmicb.2024.1298432 (PMC11148242; doi:10.3389/fmicb.2024.1298432)
Supplement: Supplementary file 1 [file Data_Sheet_1.docx]

**Supplementary Table 1** | Etiology of CKD for each included patient

| HD patients | Diabetes | Hypertension | Goodpasture disease | Polykystosis | Other* |
| --- | --- | --- | --- | --- | --- |
| 1 | x | x |  |  |  |
| 2 |  | x |  |  |  |
| 3 |  | x |  |  |  |
| 4 |  | x |  |  |  |
| 5 |  |  |  | x |  |
| 6 |  | x |  |  | x |
| 7 |  |  |  | x |  |
| 8 | x |  |  |  |  |
| 9 | x | x | x |  |  |
| 10 | x | x |  |  |  |
| 11 | x | x |  |  |  |
| 12 | x | x |  |  |  |
| 13 |  | x |  |  |  |
| 14 |  |  |  |  | x |
| 15 |  |  |  | x |  |
| 16 |  | x |  |  |  |
| 17 | x | x |  |  |  |
| 18 |  | x |  |  |  |
| 19 | x | x |  |  |  |
| 20 | x | x |  |  |  |
| 21 | x | x |  |  |  |
| 22 |  | x |  |  |  |
| NHD patients | Diabetes | Hypertension | Goodpasture disease | Polykystosis | Other* |
| 1 | x | x |  |  |  |
| 2 |  | x |  |  |  |
| 3 |  | x |  |  |  |
| 4 |  | x |  |  |  |
| 5 |  | x |  |  |  |
| 6 | x | x |  |  |  |
| 7 |  |  |  | x |  |
| 8 |  |  |  | x |  |
| 9 |  | x |  |  |  |
| 10 | x | x |  |  | x |
| 11 |  | x |  |  |  |

*Other: IgA nephropathy, monoclonal gammopathy

**Supplementary Table 2** | Treatments for diabetes and hypertension for each patient affected by these conditions

| HD patients | Diabetes | Hypertension |
| --- | --- | --- |
| 1 | Insulin  Linagliptin | Calcium channel inhibitor  ACE inhibitor  B-blocker  Loop diuretic  Centrally-acting symptholytic |
| 2 |  | B-blocker |
| 3 |  | Calcium channel inhibitor  B-blocker  Thiazide diuretic, loop diuretic |
| 4 |  | Loop diuretic |
| 6 |  | Loop diuretic |
| 8 | Insulin |  |
| 9 | - | Calcium channel inhibitor  B-blocker  Loop diuretic |
| 10 | Insulin | - |
| 11 | Insulin | B-blocker  Loop diuretic |
| 12 | Insulin | B-blocker  Loop diuretic |
| 13 |  | Loop diuretic |
| 16 |  | B-blocker |
| 17 | Insulin  Linagliptin | Calcium channel inhibitor  Angiotensin II receptor blocker  B-blocker  Loop diuretic |
| 18 |  | Calcium channel inhibitor  Loop diuretic |
| 19 | - | B-blocker  Loop diuretic |
| 20 | - | ACE inhibitor  B-blocker  Loop diuretic  Centrally-acting symptholytic |
| 21 | Insulin | Calcium channel inhibitor  B-blocker |
| 22 |  | Calcium channel inhibitor  ACE inhibitor  B-blocker  Loop diuretic  Clonidine chlorhydrate |
| NHD patients | Diabetes | Hypertension |
| 1 | Insulin  Linagliptin | Calcium channel inhibitor  B-blocker |
| 2 |  | Calcium channel inhibitor |
| 3 |  | B-blocker  Centrally-acting symptholytic |
| 4 |  | B-blocker |
| 5 |  | Calcium channel inhibitor  B-blocker  Loop diuretic |
| 6 | Insulin  Liraglutide | Angiotensin II receptor blocker  B-blocker  Thiazide diuretic, loop diuretic, potassim-sparing diuretic |
| 9 |  | Calcium channel inhibitor  Angiotensin II receptor blocker |
| 10 | Metformin | B-blocker  Loop diuretic |
| 11 |  | Calcium channel inhibitor  B-blocker |

**Supplementary Table 3** | Body composition and handgrip strength by sex

|  | HD patients | NHD patients | Healthy volunteers | p* |
| --- | --- | --- | --- | --- |
| **Female** |  |  |  |  |
| N | 6 (50.0%) | 3 (25.0%) | 3 (25.0%) |  |
| Height (cm) | 155.8 (13.2) | 151.6 (24.5) | 164.8 (2.3) | 0.4693 |
| Body weight (kg) | 69.3 (30.2) | 67.5 (10.8) | 78.7 (15.8) | 0.8042 |
| Body mass index (kg/m^2^) | 28.8 (12.6) | 29.4 (14.1) | 29.3 (6.1) | 0.8093 |
| Soft lean mass (kg) | 38.0 (15.2) | 43.0 (3.0) | 44.4 (8.4) | 0.6144 |
| Bone mineral content (kg) | 1.7 (0.3) | 1.6 (0.5) | 2.2 (0.2) | 0.1406 |
| Fat mass (kg) | 30.4 (14.0) | 25.8 (13.1) | 28.9 (11.7) | 0.8093 |
| Lean mass index (kg/m^2^) | 15.9 (6.6) | 18.1 (5.2) | 16.8 (3.0) | 0.6939 |
| Fat mass index (kg/m^2^) | 12.4 (6.7) | 11.2 (9.2) | 10.6 (4.6) | 0.8910 |
| Handgrip strength (kg) | 20.0 (6.0) | 16.0 (8.0) | 28.0 (8.0) | 0.0435 |
| **Male** |  |  |  |  |
| N | 16 (50.0%) | 8 (25.0%) | 8 (25.0%) |  |
| Height (cm) | 170.0 (11.8) | 173.1 (12.3) | 174.1 (3.2) | 0.5503 |
| Body weight (kg) | 89.3 (22.1) | 79.6 (12.0) | 78.2 (17.5) | 0.2191 |
| Body mass index (kg/m^2^) | 29.9 (4.6) | 24.9 (4.8) | 25.7 (2.1) | 0.0080 |
| Soft lean mass (kg) | 58.8 (14.9) | 55.1 (9.5) | 55.8 (11.4) | 0.8118 |
| Bone mineral content (kg) | 2.5 (0.5) | 2.3 (0.8) | 2.5 (0.7) | 0.9471 |
| Fat mass (kg) | 26.3 (9.4) | 22.9 (8.1) | 23.1 (9.5) | 0.1376 |
| Lean mass index (kg/m^2^) | 19.2 (3.4) | 17.1 (1.5) | 18.1 (2.3) | 0.1711 |
| Fat mass index (kg/m^2^) | 9.4 (2.9) | 7.5 (2.5) | 7.6 (2.3) | 0.0356 |
| Handgrip strength (kg) | 29.0 (11.0) | 36.5 (7.5) | 39.0 (8.5) | 0.0317 |

*Kruskal-Wallis H tests, Chi-squared corrected for ties with 2 degrees of freedom. With the Benjamini–Hochberg method, significance was corrected to p*<*0.026.

**Supplementary Table 4** | Bacterial taxa differentially abundant between the hemodialysis (HD) patients, non-hemodialysed patients with chronic kidney disease (NHD) and healthy volunteers (HV). Included are results with an uncorrected *p*-value <0.01, as determined by MaAsLin2. The MaAsLin2 coefficient scale is given below the plot.

| **Rank** | **Taxon** | |  | **HD  vs NHD** | **HD vs HV** | **NHD vs HV** |
| --- | --- | --- | --- | --- | --- | --- |
| Phylum |  | Bacteroidetes | | -0.8 |  |  |
| Class | Firmicutes | Tissierellia | |  | 2.5 |  |
|  | Bacteroidetes | Bacteroidia | |  | -0.8 |  |
| Order | Firmicutes;Tissierellia | Tissierellales | |  | 2.5 |  |
|  | Bacteroidetes;Bacteroidia | Bacteroidales | |  | -0.8 |  |
| Family | Firmicutes;Clostridia;Clostridiales | Eubacteriaceae | |  | 2.7 | 3.4 |
|  | Firmicutes;Tissierellia;Tissierellales | Peptoniphilaceae | |  | 2.5 |  |
| Genus | Firmicutes;Clostridia;Clostridiales;Christensenellaceae | PAC002510_g | |  | 2.7 | 3.1 |
|  | Firmicutes;Clostridia;Clostridiales;Eubacteriaceae | Anaerofustis | |  | 2.0 | 2.9 |
|  | Firmicutes;Clostridia;Clostridiales;Ruminococcaceae | Ruthenibacterium | |  | 1.9 | 2.1 |
|  | Firmicutes;Clostridia;Clostridiales;Lachnospiraceae | Clostridium_g21 | |  | 3.0 |  |
|  | Firmicutes;Clostridia;Clostridiales;Mogibacterium_f | Emergencia | |  | 2.7 |  |
|  | Firmicutes;Clostridia;Clostridiales;Lachnospiraceae | Eubacterium_g4 | |  | -1.9 | -1.8 |
|  | Firmicutes;Clostridia;Clostridiales;Lachnospiraceae | PAC001177_g | |  | -3.6 | -4.6 |
|  | Bacteroidetes;Bacteroidia;Bacteroidales;Barnesiellaceae | Coprobacter | |  | -3.0 |  |
|  | Firmicutes;Clostridia;Clostridiales;Lachnospiraceae | PAC000196_g | |  | -1.7 |  |
|  | Firmicutes;Clostridia;Clostridiales;Lachnospiraceae | PAC000740_g | |  | -4.7 |  |
|  | Firmicutes;Clostridia;Clostridiales;Lachnospiraceae | Ruminococcus_g5 | | 2.9 | 3.8 |  |
|  | Firmicutes;Erysipelotrichi;Erysipelotrichales;Erysipelotrichaceae | Bulleidia | | 1.3 |  |  |
|  | Proteobacteria;Betaproteobacteria;Burkholderiales;Sutterellaceae | Parasutterella | | -2.7 | -3.8 |  |
|  | Firmicutes;Clostridia;Clostridiales;Ruminococcaceae | PAC001402_g | | -1.0 |  |  |
|  | Lentisphaerae;Lentisphaeria;Victivallales;Victivallaceae | Victivallis | | -2.3 |  |  |
|  | Firmicutes;Clostridia;Clostridiales;Ruminococcaceae | PAC000742_g | | -2.1 |  | 1.8 |
|  | Firmicutes;Clostridia;Clostridiales;Ruminococcaceae | Caproiciproducens | |  |  | 2.0 |
| Species | Firmicutes;Clostridia;Clostridiales;Eubacteriaceae;Anaerofustis | Anaerofustis stercorihominis | |  | 2.0 | 2.9 |
|  | Firmicutes;Bacilli;Lactobacillales;Streptococcaceae;Streptococcus | Streptococcus anginosus | |  | 3.0 |  |
|  | Firmicutes;Clostridia;Clostridiales;Christensenellaceae;PAC001207_g | PAC001293_s | |  | 3.1 |  |
|  | Firmicutes;Clostridia;Clostridiales;Lachnospiraceae;Clostridium_g21 | Clostridium scindens | |  | 3.0 |  |
|  | Firmicutes;Clostridia;Clostridiales;Lachnospiraceae;Clostridium_g24 | Clostridium aldenense | |  | 2.9 |  |
|  | Firmicutes;Clostridia;Clostridiales;Lachnospiraceae;Eisenbergiella | Eisenbergiella tayi | |  | 2.5 |  |
|  | Bacteroidetes;Bacteroidia;Bacteroidales;Bacteroidaceae;Bacteroides | LT707007_s | |  | -1.9 | -1.4 |
|  | Bacteroidetes;Bacteroidia;Bacteroidales;Barnesiellaceae;Coprobacter | Coprobacter secundus | |  | -1.9 | -1.8 |
|  | Firmicutes;Clostridia;Clostridiales;Lachnospiraceae;Eubacterium_g4 | PAC001167_s | |  | -3.7 | -3.8 |
|  | Firmicutes;Clostridia;Clostridiales;Lachnospiraceae;PAC000196_g | PAC000196_s | |  | -3.0 | -3.2 |
|  | Firmicutes;Clostridia;Clostridiales;Lachnospiraceae;PAC000692_g | PAC001467_s | |  | -2.3 | -2.6 |
|  | Firmicutes;Clostridia;Clostridiales;Lachnospiraceae;PAC001138_g | PAC001138_s | |  | -3.0 | -3.2 |
|  | Firmicutes;Clostridia;Clostridiales;Lachnospiraceae;PAC001177_g | PAC001177_s | |  | -3.6 | -4.6 |
|  | Firmicutes;Clostridia;Clostridiales;Ruminococcaceae;Oscillibacter | PAC001037_s | |  | -3.2 | -3.9 |
|  | Firmicutes;Clostridia;Clostridiales;Ruminococcaceae;Oscillibacter | PAC001316_s | |  | -2.5 | -2.8 |
|  | Bacteroidetes;Bacteroidia;Bacteroidales;Barnesiellaceae;Coprobacter | Coprobacter_unclassified | |  | -3.3 |  |
|  | Firmicutes;Clostridia;Clostridiales;Lachnospiraceae;PAC000740_g | PAC000740_s | |  | -4.7 |  |
|  | Actinobacteria;Actinobacteria_c;Bifidobacteriales;Bifidobacteriaceae;Bifidobacterium | Bifidobacterium dentium | | 2.3 | 3.5 |  |
|  | Firmicutes;Clostridia;Clostridiales;Lachnospiraceae;Ruminococcus_g5 | Ruminococcus gnavus | | 2.9 | 3.8 |  |
|  | Firmicutes;Bacilli;Lactobacillales;Lactobacillaceae;Lactobacillus | Lactobacillus paracasei | | 2.8 |  |  |
|  | Firmicutes;Erysipelotrichi;Erysipelotrichales;Erysipelotrichaceae;Bulleidia | Solobacterium moorei | | 1.3 |  |  |
|  | Firmicutes;Clostridia;Clostridiales;Lachnospiraceae;PAC000196_g | PAC001335_s | | -1.0 | -1.3 |  |
|  | Firmicutes;Clostridia;Clostridiales;Lachnospiraceae;PAC001043_g | PAC001043_s | | -2.4 | -3.1 |  |
|  | Firmicutes;Clostridia;Clostridiales;Ruminococcaceae;Subdoligranulum | Subdoligranulum_unclassified | | -2.7 | -3.9 |  |
|  | Firmicutes;Clostridia;Clostridiales;Lachnospiraceae;Eubacterium_g5 | Eubacterium_g5_unclassified | | -1.3 |  |  |
|  | Firmicutes;Clostridia;Clostridiales;Ruminococcaceae;PAC001402_g | PAC001402_g_unclassified | | -1.0 |  |  |
|  | Bacteroidetes;Bacteroidia;Bacteroidales;Rikenellaceae;Alistipes | PAC001424_s | | -4.2 |  | 2.8 |
|  | Firmicutes;Clostridia;Clostridiales;Christensenellaceae;Christensenella | PAC002297_s | | -1.2 |  | 1.5 |
|  | Firmicutes;Clostridia;Clostridiales;Lachnospiraceae;PAC001270_g | PAC001270_g_unclassified | | -1.5 |  | 1.2 |
|  | Firmicutes;Clostridia;Clostridiales;Ruminococcaceae;Caproiciproducens | HQ780956_s | | -1.8 |  | 2.3 |
|  | Firmicutes;Clostridia;Clostridiales;Ruminococcaceae;PAC000742_g | PAC000742_s | | -2.1 |  | 1.8 |
|  | Firmicutes;Clostridia;Clostridiales;Ruminococcaceae;Pseudoflavonifractor | NFKI_s | | -1.8 |  | 2.9 |
|  | Firmicutes;Negativicutes;Veillonellales;Veillonellaceae;Dialister | Dialister_invisus | | -4.5 |  | 6.0 |
|  | Firmicutes;Clostridia;Clostridiales;Christensenellaceae;PAC001217_g | PAC001434_s | |  |  | 2.0 |
|  | Firmicutes;Clostridia;Clostridiales;Ruminococcaceae;Acutalibacter | PAC002394_s | |  |  | 3.0 |
|  | Firmicutes;Erysipelotrichi;Erysipelotrichales;Erysipelotrichaceae;Coprobacillus | Coprobacillus cateniformis | |  |  | 3.2 |
| zOTU | Firmicutes;Clostridia;Clostridiales;Ruminococcaceae;Ruminococcaceae_uncl;Ruminococcaceae_unclassified | zOTU662 | |  | 1.4 | 2.7 |
|  | Actinobacteria;Coriobacteriia;Coriobacteriales;Coriobacteriaceae;Eggerthella;Eggerthella lenta | zOTU133 | |  | 3.2 |  |
|  | Firmicutes;Bacilli;Lactobacillales;Streptococcaceae;Streptococcus;Streptococcus_anginosus | zOTU469 | |  | 2.7 |  |
|  | Firmicutes;Clostridia;Clostridiales;Lachnospiraceae;Clostridium_g21;Clostridium scindens | zOTU144 | |  | 3.0 |  |
|  | Firmicutes;Clostridia;Clostridiales;Lachnospiraceae;Clostridium_g24;Clostridium aldenense | zOTU879 | |  | 2.1 |  |
|  | Firmicutes;Clostridia;Clostridiales;Lachnospiraceae;Eisenbergiella;Eisenbergiella tayi | zOTU533 | |  | 2.5 |  |
|  | Firmicutes;Clostridia;Clostridiales;Ruminococcaceae;Sporobacter;Sporobacter_unclassified | zOTU1088 | |  | 1.6 |  |
|  | Bacteroidetes;Bacteroidia;Bacteroidales;Bacteroidaceae;Bacteroides;LT707007_s | zOTU3117 | |  | -1.3 | -1.1 |
|  | Bacteroidetes;Bacteroidia;Bacteroidales;Barnesiellaceae;Coprobacter;Coprobacter secundus | zOTU1051 | |  | -1.9 | -1.8 |
|  | Firmicutes;Clostridia;Clostridiales;Lachnospiraceae;Eubacterium_g4;PAC001167_s | zOTU234 | |  | -3.7 | -3.8 |
|  | Firmicutes;Clostridia;Clostridiales;Lachnospiraceae;PAC000196_g;PAC000196_s | zOTU610 | |  | -3.0 | -3.2 |
|  | Firmicutes;Clostridia;Clostridiales;Lachnospiraceae;PAC000692_g;PAC001467_s | zOTU797 | |  | -2.3 | -2.6 |
|  | Firmicutes;Clostridia;Clostridiales;Lachnospiraceae;PAC001138_g;PAC001138_s | zOTU379 | |  | -3.0 | -3.2 |
|  | Firmicutes;Clostridia;Clostridiales;Lachnospiraceae;PAC001177_g;PAC001177_s | zOTU98 | |  | -4.0 | -5.2 |
|  | Firmicutes;Clostridia;Clostridiales;Ruminococcaceae;Oscillibacter;PAC001037_s | zOTU298 | |  | -1.9 | -2.2 |
|  | Firmicutes;Clostridia;Clostridiales;Ruminococcaceae;Oscillibacter;PAC001037_s | zOTU950 | |  | -1.9 | -2.8 |
|  | Bacteroidetes;Bacteroidia;Bacteroidales;Barnesiellaceae;Coprobacter;Coprobacter_unclassified | zOTU694 | |  | -2.8 |  |
|  | Bacteroidetes;Bacteroidia;Bacteroidales;Prevotellaceae;Paraprevotella;Paraprevotella clara | zOTU287 | |  | -4.0 |  |
|  | Firmicutes;Clostridia;Clostridiales;Lachnospiraceae;Lachnospiraceae_uncl;Lachnospiraceae_unclassified | zOTU1234 | |  | -1.4 |  |
|  | Firmicutes;Clostridia;Clostridiales;Lachnospiraceae;Lachnospiraceae_uncl;Lachnospiraceae_unclassified | zOTU556 | |  | -2.2 |  |
|  | Firmicutes;Clostridia;Clostridiales;Lachnospiraceae;PAC000740_g;PAC000740_s | zOTU86 | |  | -4.0 |  |
|  | Firmicutes;Clostridia;Clostridiales;Ruminococcaceae;Pseudoflavonifractor;EF403010_s | zOTU2539 | |  | -1.0 |  |
|  | Firmicutes;Bacilli;Lactobacillales;Streptococcaceae;Streptococcus;Streptococcus_unclassified | zOTU388 | | 2.0 | 3.7 |  |
|  | Firmicutes;Clostridia;Clostridiales;Lachnospiraceae;Ruminococcus_g5;Ruminococcus gnavus | zOTU35 | | 2.9 | 3.8 |  |
|  | Firmicutes;Bacilli;Lactobacillales;Lactobacillaceae;Lactobacillus;Lactobacillus paracasei | zOTU363 | | 2.8 |  |  |
|  | Firmicutes;Erysipelotrichi;Erysipelotrichales;Erysipelotrichaceae;Bulleidia;Solobacterium moorei | zOTU1045 | | 1.3 |  |  |
|  | Firmicutes;Clostridia;Clostridiales;Lachnospiraceae;PAC000196_g;PAC001335_s | zOTU282 | | -1.0 | -1.3 |  |
|  | Firmicutes;Clostridia;Clostridiales;Lachnospiraceae;PAC001043_g;PAC001043_s | zOTU405 | | -2.4 | -3.1 |  |
|  | Firmicutes;Clostridia;Clostridiales;Ruminococcaceae;Subdoligranulum;Subdoligranulum_unclassified | zOTU227 | | -2.7 | -3.9 |  |
|  | Bacteroidetes;Bacteroidia;Bacteroidales;Barnesiellaceae;Barnesiella;Barnesiella_intestinihominis | zOTU326 | | -1.5 |  |  |
|  | Bacteroidetes;Bacteroidia;Bacteroidales;Rikenellaceae;Alistipes;PAC001424_s | zOTU407 | | -3.6 |  |  |
|  | Firmicutes;Clostridia;Clostridiales;Ruminococcaceae;Caproiciproducens;Caproiciproducens_unclassified | zOTU1905 | | -1.5 |  |  |
|  | Firmicutes;Clostridia;Clostridiales;Eubacteriaceae;Anaerofustis;Anaerofustis stercorihominis | zOTU1239 | | -1.9 |  | 2.5 |
|  | Firmicutes;Clostridia;Clostridiales;Ruminococcaceae;Caproiciproducens;HQ780956_s | zOTU1194 | | -2.0 |  | 2.1 |
|  | Firmicutes;Clostridia;Clostridiales;Ruminococcaceae;Pseudoflavonifractor;FJ505502_s | zOTU1685 | | -1.1 |  | 1.4 |
|  | Firmicutes;Negativicutes;Veillonellales;Veillonellaceae;Dialister;Dialister invisus | zOTU73 | | -4.5 |  | 6.0 |
|  | Firmicutes;Clostridia;Clostridiales;Christensenellaceae;PAC001217_g;PAC001434_s | zOTU994 | |  |  | 2.0 |
|  | Firmicutes;Clostridia;Clostridiales;Lachnospiraceae;Dorea;Dorea massiliensis | zOTU179 | |  |  | 3.9 |
|  | Firmicutes;Clostridia;Clostridiales;Ruminococcaceae;Acutalibacter;PAC002394_s | zOTU775 | |  |  | 3.0 |
|  | Firmicutes;Clostridia;Clostridiales;Ruminococcaceae;Pseudoflavonifractor;NFKI_s | zOTU621 | |  |  | 3.1 |
|  | Firmicutes;Erysipelotrichi;Erysipelotrichales;Erysipelotrichaceae;Coprobacillus;Coprobacillus cateniformis | zOTU660 | |  |  | 3.2 |

-6

0

6

**Supplementary Table 5** | Correlations between the significant variables identified in Table 3 (height, soft lean body mass, bone mineral content, resting energy expenditure, handgrip strength, plasma albumin level, plasma Tumor necrosis factor-α level) and zOTUs. Displayed are correlations with a Spearman coefficient greater than 0.5 (red) and less than –0.5 (blue).

| **zOTU ID** | **Taxonomy** | **Variable** | **Correlation coefficient*** |
| --- | --- | --- | --- |
| zOTU286 | Bacteroidetes;Bacteroidia;Bacteroidales;Porphyromonadaceae;Parabacteroides;Parabacteroides_unclassified | Resting energy expenditure (kcal) | 0.546 |
| zOTU343 | Firmicutes;Clostridia;Clostridiales;Lachnospiraceae;Lachnospira;PAC001142_s |  | 0.579 |
| zOTU389 | Firmicutes;Clostridia;Clostridiales;Lachnospiraceae;Lachnospiraceae_unclassified;Lachnospiraceae_unclassified |  | 0.526 |
| zOTU98 | Firmicutes;Clostridia;Clostridiales;Lachnospiraceae;PAC001177_g;PAC001177_s |  | 0.703 |
| zOTU314 | Firmicutes;Clostridia;Clostridiales;Lachnospiraceae;PAC001269_g;PAC001269_s |  | 0.545 |
| zOTU869 | Firmicutes;Clostridia;Clostridiales;Mogibacterium_f;PAC001609_g;PAC001609_g_unclassified |  | 0.520 |
| zOTU2510 | Firmicutes;Clostridia;Clostridiales;Peptococcaceae;PAC001401_g;PAC001401_s |  | 0.593 |
| zOTU950 | Firmicutes;Clostridia;Clostridiales;Ruminococcaceae;Oscillibacter;PAC001037_s |  | 0.541 |
| zOTU889 | Firmicutes;Clostridia;Clostridiales;Ruminococcaceae;Agathobaculum;Agathobaculum_unclassified |  | 0.509 |
| zOTU90 | Firmicutes;Clostridia;Clostridiales;Ruminococcaceae;PAC000661_g;PAC001248_s |  | 0.506 |
| zOTU3553 | Actinobacteria;Actinobacteria_c;Actinomycetales;Actinomycetaceae;Actinomyces;Actinomyces israelii | Tumor necrosis factor-α (pg/ml) | 0.536 |
| zOTU1160 | Actinobacteria;Actinobacteria_c;Actinomycetales;Actinomycetaceae;Actinomyces;Actinomyces_unclassified |  | 0.533 |
| zOTU784 | Actinobacteria;Coriobacteriia;Coriobacteriales;Coriobacteriaceae;Gordonibacter;Gordonibacter faecihominis |  | 0.534 |
| zOTU388 | Firmicutes;Bacilli;Lactobacillales;Streptococcaceae;Streptococcus;Streptococcus_unclassified |  | 0.617 |
| zOTU536 | Actinobacteria;Coriobacteriia;Coriobacteriales;Coriobacteriaceae;AM278923_g;AM278923_s | Height (cm) | 0.566 |
| zOTU250 | Actinobacteria;Coriobacteriia;Coriobacteriales;Coriobacteriaceae;Slackia;Slackia isoflavoniconvertens |  | 0.520 |
| zOTU313 | Firmicutes;Clostridia;Clostridiales;Lachnospiraceae;PAC001200_g;PAC001200_s |  | 0.587 |
| zOTU349 | Firmicutes;Clostridia;Clostridiales;Ruminococcaceae;Eubacterium_g8;Eubacterium siraeum |  | 0.524 |
| zOTU446 | Firmicutes;Clostridia;Clostridiales;Ruminococcaceae;Oscillibacter;PAC001290_s |  | 0.520 |
| zOTU345 | Bacteroidetes;Bacteroidia;Bacteroidales;Porphyromonadaceae;Parabacteroides;PAC001783_s | Albumin (g/l) | 0.512 |
| zOTU8 | Firmicutes;Clostridia;Clostridiales;Ruminococcaceae;Faecalibacterium;NMTZ_s |  | 0.530 |
| zOTU697 | Firmicutes;Clostridia;Clostridiales;Lachnospiraceae;PAC001283_g;PAC001283_s | Bone mineral content (kg) | 0.527 |
| zOTU314 | Firmicutes;Clostridia;Clostridiales;Lachnospiraceae;PAC001269_g;PAC001269_s | Soft lean body mass (kg) | 0.507 |
| zOTU211 | Firmicutes;Clostridia;Clostridiales;Lachnospiraceae;Clostridium_g24;Clostridium bolteae | Resting energy expenditure (kcal) | -0.510 |
| zOTU541 | Firmicutes;Clostridia;Clostridiales;Lachnospiraceae;Clostridium_g24;Clostridium lavalense |  | -0.512 |
| zOTU234 | Firmicutes;Clostridia;Clostridiales;Lachnospiraceae;Eubacterium_g4;PAC001167_s | Tumor necrosis factor-α (pg/ml) | -0.561 |
| zOTU559 | Firmicutes;Clostridia;Clostridiales;Ruminococcaceae;Sporobacter;Sporobacter_unclassified |  | -0.510 |
| zOTU1160 | Actinobacteria;Actinobacteria_c;Actinomycetales;Actinomycetaceae;Actinomyces;Actinomyces_unclassified | Albumin (g/l) | -0.536 |
| zOTU363 | Firmicutes;Bacilli;Lactobacillales;Lactobacillaceae;Lactobacillus;Lactobacillus paracasei |  | -0.519 |
| zOTU211 | Firmicutes;Clostridia;Clostridiales;Lachnospiraceae;Clostridium_g24;Clostridium bolteae | Soft lean body mass (kg) | -0.593 |
| zOTU801 | Firmicutes;Clostridia;Clostridiales;Lachnospiraceae;Clostridium_g24;Clostridium citroniae |  | -0.620 |
| zOTU355 | Firmicutes;Clostridia;Clostridiales;Lachnospiraceae;Clostridium_g35;Clostridium symbiosum |  | -0.509 |

*Spearman’s test
